# Long-term safety, tolerability and efficacy of apomorphine sublingual film in patients with Parkinson’s disease complicated by OFF episodes: a phase 3, open-label study

**DOI:** 10.1007/s00415-024-12323-2

**Published:** 2024-03-28

**Authors:** Jan Kassubek, Stewart A. Factor, Ernest Balaguer, Johannes Schwarz, K. Ray Chaudhuri, Stuart H. Isaacson, Stacy Wu, Carmen Denecke Muhr, Jaime Kulisevsky

**Affiliations:** 1 Department of Neurology, University Hospital Ulm, Oberer Eselsberg 45, 89081 Ulm, Germany; 2 German Centre for Neurodegenerative Diseases, Ulm, Germany; 3 Jean and Paul Amos Parkinson’s Disease and Movement Disorder Program, Emory University School of Medicine, Atlanta, GA USA; 4 Hospital Universitari General de Catalunya, Barcelona, Spain; 5 Department of Geriatrics, Kreisklinik Ebersberg, Ebersberg, Germany; 6 Department of Neurosciences, Institute of Psychiatry, Psychology and Neuroscience and Parkinson’s Foundation Centre of Excellence, King’s College Hospital, King’s College London, London, UK; 7 Parkinson’s Disease and Movement Disorders Center of Boca Raton, Boca Raton, FL USA; 8 Sumitomo Pharma America, Inc., Marlborough, MA USA; 9 BIAL-Portela & Ca S.A., Porto, Portugal; 10 Hospital de la Santa Creu i Sant Pau, Barcelona, Spain; 11 Universitat Autònoma de Barcelona and CIBERNED, Madrid, Spain

**Keywords:** Apomorphine sublingual film, Motor fluctuations, OFF episodes, Parkinson’s disease

## Abstract

**Background:**

Apomorphine sublingual film (SL-APO) is an on-demand treatment for OFF episodes in patients with Parkinson’s disease (PD).

**Objective:**

To assess the long-term (≥ 3 years) safety/tolerability and efficacy of SL-APO.

**Methods:**

Study CTH-301 (http://www.clinicaltrials.gov NCT02542696; registered 2015-09-03) was a phase 3, multicentre, open-label study of SL-APO in PD patients with motor fluctuations, comprised of a dose-titration and long-term safety phase. All participants received SL-APO. The primary endpoint was safety/tolerability (treatment-emergent adverse events [TEAEs]) during the long-term safety phase. Efficacy assessments included the Movement Disorder Society-Unified Parkinson’s Disease Rating Scale (MDS-UPDRS) part III (motor examination), assessed at weeks 24, 36 and 48 during the first year of the long-term safety phase.

**Results:**

496 patients were included and 120 (24.2%) completed the long-term safety phase. Mean duration of SL-APO exposure was 294.3 days. TEAEs related to study drug were experienced by 65.3% of patients (most common: nausea [6.0%], stomatitis [1.8%], lip swelling [1.8%], dizziness [1.6%], oral mucosal erythema [1.6%], mouth ulceration [1.6%]). TEAEs leading to study drug withdrawal were experienced by 34.0% of patients (most common: nausea [5.4%], lip swelling [4.5%], mouth ulceration [2.6%], stomatitis [2.3%]). A clinically meaningful reduction in MDS-UPDRS part III score was observed as soon as 15 min following administration of SL-APO, with peak effects observed approximately 30 min post-dose and sustained up to 90 min post-dose; results were consistent over 48 weeks.

**Conclusions:**

SL-APO was generally well tolerated and efficacious over the long term as an on-demand treatment for OFF episodes in patients with PD.

**Supplementary Information:**

The online version contains supplementary material available at 10.1007/s00415-024-12323-2.

## Introduction

Parkinson’s disease (PD) is the second most common neurodegenerative disorder (after Alzheimer’s disease), affecting 2‒3% of those aged ≥ 65 years [1, 2]. The key neurochemical feature resulting in cardinal motor symptoms is dopamine deficiency caused by neuronal degeneration in the substantia nigra with α-synuclein aggregation forming Lewy bodies [2]. Levodopa continues to be the most effective pharmacological treatment for PD [3–5]. It effectively controls motor symptoms in the early stages of disease, but with progression the therapeutic window narrows and the response becomes less predictable with shorter more variable duration of effect, resulting in increasing motor and nonmotor fluctuations, which include OFF episodes of variable duration and severity [6–8]. OFF episodes are often associated with worsened non-motor symptoms, such as pain, anxiety and depression, and can impact the quality of life of patients with PD, causing disability and an inability to participate in social activities [5]. OFF episodes are experienced by approximately 50% of patients after 5 years and most patients by 9 years [8]. Shortening of the clinical benefit of levodopa and the associated emergence of OFF episodes are thought to be related to progressive loss of dopaminergic neurons and their ability to store and release levodopa-derived dopamine over a sustained time period [9]. However, the emergence of OFF episodes is also likely to be influenced by factors that impact the optimal delivery of oral levodopa, such as: impaired swallowing and oesophageal dysmotility, including dysphagia for medication [10] and gastrointestinal mobility disturbances (which are a very common symptom of patients with PD [11]), associated with resulting gastrointestinal bacterial overgrowth reducing absorption; extensive peripheral breakdown of levodopa by aromatic L-amino acid decarboxylase and catechol-O-methyl transferase; *Helicobacter pylori* infection [12, 13]; a protein-rich diet [14]; and competition with amino acids for transport into the brain [9, 15–18]. Several ‘on-demand’ therapies have been developed for the treatment of OFF episodes that specifically address the challenges associated with absorption via the gut, including levodopa inhalation powder (CVT-301) and a subcutaneous injection formulation of the non-ergoline dopamine agonist apomorphine [8, 19–22]. Apomorphine sublingual film (SL-APO) was developed to address the limitations associated with subcutaneous apomorphine (such as skin irritation and “needle phobia” [23]). It was designed to be placed under the tongue and deliver apomorphine systemically via absorption from the oral cavity mucosa [24, 25]. SL-APO was shown to be an effective and generally well-tolerated on-demand treatment for OFF episodes in patients with PD when assessed over a 12-week maintenance period in a phase 3, randomised, double-blind, placebo-controlled, parallel-group trial (CTH-300) [24]. The objective of the current study (CTH-301) was to assess the safety, tolerability and efficacy of SL-APO when used as an on-demand treatment for OFF episodes over the long term.

## Methods

### Study design

**Fig. 1:**
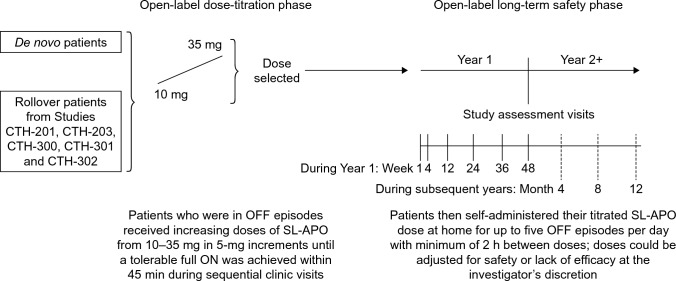
Study design. *SL-APO* apomorphine sublingual film

Study CTH-301 was a phase 3, multicentre, non-randomised, open-label study of SL-APO in PD patients with motor fluctuations, comprised of a dose-titration and long-term safety phase (Fig. 1). The study included both patients who had not previously participated in a study with SL-APO (defined as ‘de novo patients’; i.e. patients de novo to SL-APO treatment, not de novo PD patients) as well as ‘rollover patients’ who had completed SL-APO studies CTH-201 (phase 2) [26], CTH-203 (phase 2) [27], CTH-300 (phase 3) [24], or CTH-302 (phase 3) [28].

All subjects received SL-APO, with dose determination and adjustments based on efficacy, safety and tolerability. For de novo patients, SL-APO dosing was determined during a 3-week titration phase with titration in 5-mg increments over the dose range 10‒35 mg (35 mg administered as 20 mg followed by 15 mg). Patients seen in a practically defined OFF state (i.e. where medication was withheld for approximately 12 h) received increasing doses of SL-APO in sequential clinic visits until a tolerable full ON was achieved within 45 min of the dose. Initially, rollover patients also underwent SL-APO dose titration as described above. After a protocol amendment, rollover patients were assigned the same SL-APO dose they received in the previous study without needing to go through dose titration again. During the long-term safety phase, patients received SL-APO for the treatment of up to five OFF episodes per day. Patients self-administered SL-APO at home with a minimum of 2 h between doses. Patients attended clinic visits for screening and titration and then at weeks 4, 12, 24, 36 and 48 during the first year. Patients were also contacted by telephone at weeks 2, 8, 18, 30 and 42 to collect safety information and changes to concomitant medication. During subsequent years, patients attended clinic visits at months 4, 8 and 12 to undergo safety assessments, and were contacted by telephone at months 2, 6 and 10 to collect safety information and changes to concomitant medication. Dose adjustments were allowed for any patient, at the investigator’s discretion. Patients could receive the adjusted dose at additional in-clinic visits and undergo efficacy and additional safety assessments, as deemed appropriate. Patients were also contacted by telephone within 3 days after dose adjustment visits to assess the effect of the dose adjustment and safety. Patients could continue in the study until the study was terminated by the sponsor, or until SL-APO became commercially available in their country. Safety and tolerability were assessed during the dose-titration phase and entire long-term safety phase.

The study was conducted in accordance with International Council for Harmonisation (ICH) Good Clinical Practice Guidance, the ethical principles that had their origin in the Declaration of Helsinki, and all applicable local law(s) and regulation(s). In accordance with ICH guidance, the protocol (including the final version of the patient informed consent form) was approved by the Institutional Review Board (IRB)/Independent Ethics Committee (IEC) before any patients were enrolled (see Supplementary Information for details of central IRBs/IECs). All patients provided written informed consent prior to participation in the study. The study is registered on ClinicalTrials.gov (NCT02542696) and EudraCT (2016-000637-43).

### Study population

Male and female patients aged ≥ 18 years were included as de novo patients in the study if they had a clinical diagnosis of idiopathic PD (consistent with United Kingdom Parkinson’s Disease Brain Bank Clinical Diagnostic Criteria), a clinically meaningful response to levodopa (as determined by the investigator), stage 1‒3 on the modified Hoehn and Yahr scale when in ON state, and a Mini-Mental State Examination score of > 25. They were also required to be receiving stable doses of levodopa/carbidopa and adjunctive PD medications for ≥ 4 weeks (≥ 8 weeks for monoamine oxidase B inhibitors) before the first screening visit, and to be experiencing at least one OFF episode per day with a total daily OFF time of ≥ 2 h. Rollover patients were included if they had completed a prior SL-APO study and if there were no major changes in concomitant PD medications from the prior study. De novo patients were excluded from participation if they had atypical or secondary parkinsonism, a major psychiatric disorder, mouth cankers/sores ≤ 30 days before the first screening visit, a history of clinically significant hallucinations in the past 6 months, or a history of clinically significant impulse control disorder(s). De novo patients were also not included if they had received previous treatment with a neurosurgical procedure for PD, intraduodenal levodopa, continuous subcutaneous apomorphine infusion, or subcutaneous apomorphine ≤ 7 days before the second screening visit, or if they were currently receiving 5-hydroxy tryptophan (serotonin) receptor antagonists, dopamine receptor antagonists (excluding quetiapine and clozapine), or dopamine-depleting agents. Rollover patients were excluded if they developed mouth cankers/sores ≤ 14 days after completing their previous SL-APO study. Additional eligibility criteria are presented in the Supplementary Information.

### Study assessments

The primary endpoint was evaluation of the safety and tolerability of SL-APO based on the incidence of treatment-emergent adverse events (TEAEs) during the long-term safety phase. The incidence of TEAEs was also assessed during the dose-titration phase.

Secondary efficacy endpoints (assessed during the first year) were the mean change in Movement Disorder Society-Unified Parkinson’s Disease Rating Scale (MDS-UPDRS) part III (motor examination) score from pre-dose to 15, 30, 60 and 90 min post-dose at weeks 24, 36 and 48 of the long-term safety phase; the percentage of patients with a self-rated full ON (during clinic visits) within 30 min post-dose at weeks 24, 36 and 48 of the long-term safety phase; and the percentage of patients with a self-rated full ON (recorded in home dosing and response diaries) within 30 min post-dose in the 2 days before clinic visits at weeks 24, 36 and 48 of the long-term safety phase. Additional efficacy assessments included: the Clinical Global Impression of Improvement (CGI-I) post-dosing and the Patient Global Impression of Improvement (PGI-I) post-dosing; and an Ease of Use Questionnaire.

Additional safety endpoints included: physical examination (including examination of the oropharyngeal cavity); vital signs; TEAEs of special interest (allergic/sensitivity response to the formulation; daytime sudden onset of sleepiness; dyskinesias; falls and injuries; hallucinations and psychotic behaviours; hypotension and orthostatic hypotension; QT prolongation and ventricular arrhythmias; stomatitis, oral ulcers and oral irritation or allergic/hypersensitivity reaction to the formulation; syncope); incidence of oropharyngeal and dopaminergic TEAEs; clinical laboratory tests (including haematology, serum chemistry and urinalysis); and the Questionnaire for Impulsive-Compulsive Disorders in PD-Rating Scale (QUIP-RS) score.

### Statistical analysis

The full analysis set (FAS) was defined as all patients who were enrolled and received at least one dose of study medication during the long-term safety phase. The FAS was used for all efficacy and safety assessments. The Safety Population was defined as all subjects who enrolled and received at least one dose of study medication. This analysis set was used for all patient listings other than patient disposition.

All data were summarised descriptively and no statistical testing was performed. Continuous variables were summarised using the number of observations (n), mean, standard deviation (SD), median, minimum and maximum. Categorical variables were summarised as frequency counts and percentages.

## Results

### Study population

**Fig. 2:**
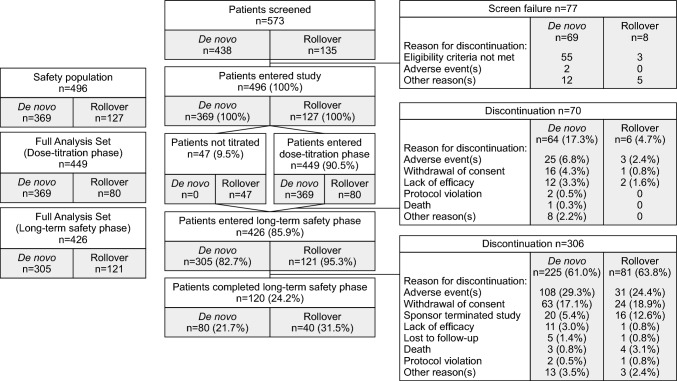
Patient disposition

A total of 573 patients were screened (de novo, n = 438; rollover, n = 135), of whom 496 entered the dose-titration phase (safety population; de novo, n = 369; rollover, n = 127), 426 continued into the long-term safety phase (FAS; de novo, n = 305; rollover, n = 121), and 120 (24.2%; de novo, n = 80; rollover, n = 40) completed the long-term safety phase (Fig. 2). Of the 127 rollover patients who entered the dose-titration phase, 47 were not titrated and 80 underwent titration. The rate of discontinuation was higher in de novo than in rollover patients, particularly during the dose-titration phase (17.3% vs. 4.7%). The majority of discontinuations occurred during the long-term safety phase, most commonly due to adverse events (AEs; 28.0%), withdrawal of patient consent (17.5%) and termination of the study by the sponsor (7.3%). One patient died during the dose-titration phase and seven patients died during the long-term safety phase, but none of these deaths was considered to be related to the study drug by the investigator.

Of the 496 patients who entered the study, 398 (80.2%) were from North America and 98 (18.2%) were from Europe. Demographic and baseline disease characteristics were generally comparable between the de novo and rollover subgroups, except that 90.0% of de novo patients were enrolled in North America, whereas rollover patients were from studies conducted in North America and Europe (52.0% from North America, 48.0% from Europe). In the total population (Table 1), the mean age was 64.4 years, 67.1% were men and 96.4% were White. The mean (SD) time since PD diagnosis was 8.7 (4.5) years and the mean (SD) time since onset of motor fluctuations was 4.5 (3.7) years. The most common types of OFF episodes were wearing-off (96.8%), delayed ON (69.2%) and morning akinesia (64.9%). The mean (SD) number of OFF episodes typically experienced per day at baseline was 3.9 (1.3) and the mean (SD) typical duration of OFF episodes was 75.3 (53.8) min. The mean MDS-UPDRS part III score in OFF state prior to levodopa administration at screening was 42.0 (14.6) and the mean (SD) total daily levodopa dose (i.e. the sum of levodopa doses reported by the patient at the first dose date) was 1097.7 (802.2) mg.

**Table 1:** Patient demographic and baseline characteristics, and use of concomitant PD medications (safety population)

	Total population N = 496
Age, years	
Mean (SD)	64.4 (8.7)
Median (min, max)	65.0 (38, 83)
Sex, n (%)	
Male	333 (67.1)
Female	163 (32.9)
Geographical region, n (%)	
North America	398 (80.2)
Europe	98 (19.8)
Race, n (%)	
White	478 (96.4)
Black or African American	10 (2.0)
Asian	4 (0.8)
American Indian or Alaska Native	1 (0.2)
Native Hawaiian or Other Pacific	1 (0.2)
Other	2 (0.4)
Time since PD diagnosis, years	
Mean (SD)	8.7 (4.5)
Median (min, max)	8.0 (0.5, 27.0)
Time since motor fluctuations started, mean (SD) years	4.5 (3.7)^a^
Type of OFF episodes experienced, n (%)	
Wearing-off	480 (96.8)
Delayed ON	343 (69.2)
Morning akinesia	322 (64.9)
Dose failure	218 (44.0)
Sudden OFF	212 (42.7)
Number of OFF episodes typically experienced/day, mean (SD)	3.9 (1.3)^a^
Typical duration of OFF episodes, mean (SD) minutes	75.3 (53.8)
Modified Hoehn and Yahr score (ON state), n (%)	
0‒1.5	23 (4.6)
2‒2.5	296 (59.7)
≥ 3	43 (8.7)
Missing	134 (27.0)
MDS-UPDRS Part III score in OFF state prior to levodopa administration at screening	
n	367
Mean (SD)	42.0 (14.6)
Median (min, max)	42.0 (11, 87)
Total daily levodopa dose, mg	1097.7 (802.2)^b^
Use of ≥ 1 concomitant PD medication,^c^ n (%)	496 (100)
Most commonly used^d^ concomitant PD medications,^c^ n (%)	
Levodopa and levodopa derivatives	496 (100)
Sinemet	438 (88.3)
Stalevo	65 (13.1)
Madopar	47 (9.5)
Dopamine agonists	315 (63.5)
Pramipexole	120 (24.2)
Ropinirole	109 (22.0)
Rotigotine	86 (17.3)
Monoamine oxidase B inhibitors	231 (46.6)
Rasagiline	164 (33.1)
Safinamide	29 (5.8)
Adamantane derivatives	122 (24.6)
Amantadine	121 (24.4)
Other dopaminergic agents	80 (16.1)
Entacapone	60 (12.1)
Opicapone	19 (3.8)

N = 496 for total population unless otherwise stated

*max* maximum, *MDS-UPDRS* Movement Disorder Society-sponsored Unified Parkinson’s Disease Rating Scale, *min* minimum, *PD* Parkinson’s disease, *SD* standard deviation

^a^N = 494

^b^N = 481

^c^Concomitant PD medications were those with a start or stop date on or after the first date of study drug dosing

^d^≥ 10% of de novo or rollover patients

### SL-APO dosing/exposure and concomitant medications

**Fig. 3:**
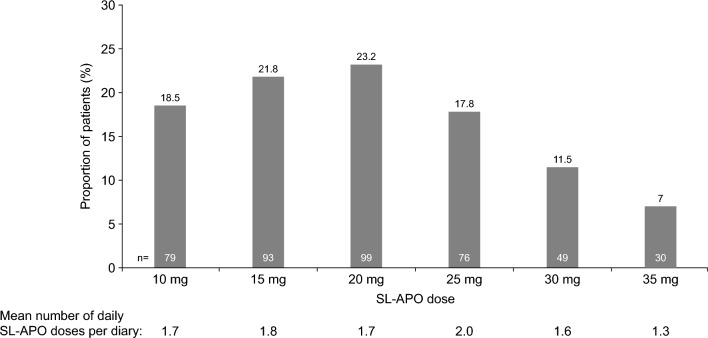
Highest dose of SL-APO received during the long-term safety phase (full analysis set). *SL-APO* apomorphine sublingual film

For most patients, the highest dose received during the dose-titration phase was equivalent to the highest dose received during the long-term safety phase. During the long-term safety phase, 63.6% of patients received a highest dose of 10‒20 mg (Fig. 3). Mean (SD) duration of exposure to SL-APO was 294.3 (312.9) days (median 169.0; range 1‒1181 days). Exposure was longer in rollover patients (mean 369.2; SD 348.9; median 177.0; range 1‒1181) than in de novo patients (mean 264.6; SD 292.7; median 168.0; range 1‒1181). The mean (SD) number of daily doses (per diary) was 1.7 (1.2) (median 1.5; range 0‒5; n = 343). The mean (SD) total daily dose of SL-APO was 34.2 (26.5) mg (median 28.1; range 0.0‒116.7; n = 343). All patients (496/496) received treatment with levodopa and levodopa derivatives, most commonly with levodopa/carbidopa (88.3%). Dopamine agonists were used by 63.5% of patients, monoamine oxidase-B inhibitors by 46.6%, and adamantane derivatives (mainly amantadine) by 24.6%. Other dopaminergic agents, such as catechol-O-methyl transferase inhibitors, were used by 16.1% of patients (entacapone 12.1%; opicapone 3.8%). The rate of discontinuation was lower in patients treated concomitantly with dopamine agonists versus those not treated with dopamine agonists, during both the dose-titration phase (10.8% [34/315] vs. 19.9% [36/181]) and long-term safety phase (60.0% [189/315] vs. 64.6% [117/181]).

### Safety/tolerability

#### TEAEs

A summary of TEAEs reported during the dose-titration phase and long-term safety phase is presented in Table 2.

**Table 2:** Summary of TEAEs during the dose-titration phase and long-term safety phase (full analysis set)

	Dose-titration phase N = 449	Long-term safety phase N = 426
Patients with any TEAE, n (%)	232 (51.7)	365 (85.7)
Total number of events, m	657	1966
Most frequently reported^a^ TEAEs, n (%)		
Nausea	67 (14.9)	91 (21.4)
Fall	4 (0.9)	44 (10.3)
Somnolence	30 (6.7)	32 (7.5)
Oral mucosal erythema	17 (3.8)	30 (7.0)
Lip swelling	0	27 (6.3)
Dizziness	27 (6.0)	27 (6.3)
Mouth ulceration	3 (0.7)	25 (5.9)
Dyskinesia	12 (2.7)	24 (5.6)
Stomatitis	2 (0.4)	23 (5.4)
Yawning	43 (9.6)	23 (5.4)
Fatigue	18 (4.0)	22 (5.2)
Headache	23 (5.1)	14 (3.3)
Patients with drug-related^b^ TEAEs, n (%)	173 (38.5)	278 (65.3)
Total number of related events, m	469	973
Most frequently reported^c^ drug-related^b^ TEAEs, m (%)^d^		
Nausea	72 (11.0)^d^	117 (6.0)^d^
Stomatitis	2 (0.3)^d^	36 (1.8)^d^
Lip swelling	0	36 (1.8)^d^
Dizziness	33 (5.0)^d^	32 (1.6)^d^
Oral mucosal erythema	6 (0.9)^d^	31 (1.6)^d^
Mouth ulceration	2 (0.3)^d^	32 (1.6)^d^
Somnolence	47 (7.2)^d^	29 (1.5)^d^
Yawning	69 (10.5)^d^	29 (1.5)^d^
Dyskinesia	13 (2.0)^d^	26 (1.3)^d^
Vomiting	7 (1.1)^d^	25 (1.3)^d^
Oral pain	0	20 (1.0)^d^
Fatigue	23 (3.5)^d^	18 (0.9)^d^
Dysgeusia	9 (1.4)^d^	15 (0.8)^d^
Headache	26 (4.0)^d^	11 (0.6)^d^
Hyperhidrosis	9 (1.4)^d^	11 (0.6)^d^
Rhinorrhoea	10 (1.5)^d^	8 (0.4)^d^
Feeling cold	11 (1.7)^d^	6 (0.3)^d^
Patients with severe^e^ TEAEs, n (%)	14 (3.1)	57 (13.4)
Severity of events, m (%)^d^		
Mild	526 (80.1)^d^	1143 (58.1)^d^
Moderate	114 (17.4)^d^	712 (36.2)^d^
Severe^e^	17 (2.6)^d^	111 (5.6)^d^
Patients with serious TEAEs, n (%)	6 (1.3)	58 (13.6)
Most frequently reported^f^ serious TEAEs, n (%)		
Pneumonia	0	5 (1.2)
Fall	0	5 (1.2)
Urinary tract infection	0	4 (0.9)
Acute myocardial infarction	0	3 (0.7)
Cardiac failure congestive	0	3 (0.7)
Patients with serious related TEAEs, n (%)	1 (0.2)	4 (0.9)
Patients with TEAEs leading to death, n (%)	1 (0.2)	7 (1.6)
Related TEAEs leading to death, n (%)	0	0
Patients with TEAEs leading to study drug withdrawal, n (%)	30 (6.7)	145 (34.0)
Most frequently reported^g^ TEAEs leading to study drug withdrawal, n (%)		
Nausea	8 (1.8)	23 (5.4)
Lip swelling	0	19 (4.5)
Mouth ulceration	0	11 (2.6)
Stomatitis	0	10 (2.3)
Patients with TEAEs of special interest, n (%)	121 (26.9)	268 (62.9)
Most frequently reported^a^ TEAEs of special interest, n (%)		
Fall	4 (0.9)	44 (10.3)
Somnolence	30 (6.7)	32 (7.5)
Oral mucosal erythema	17 (3.8)	30 (7.0)
Lip swelling	0	27 (6.3)
Dizziness	27 (6.0)	27 (6.3)
Mouth ulceration	3 (0.7)	25 (5.9)
Dyskinesia	12 (2.7)	24 (5.6)
Stomatitis	2 (0.4)	23 (5.4)
Patients with serious TEAEs of special interest, n (%)	0	31 (7.3)
Most frequently reported serious^f^ TEAEs of special interest, n (%)		
Fall	0	5 (1.2)
Acute myocardial infarction	0	3 (0.7)
Patients with TEAEs of special interest leading to study drug withdrawal, n (%)	0	106 (24.9)
Most frequently reported^g^ TEAEs of special interest leading to study drug withdrawal, n (%)		
Lip swelling	0	19 (4.5)
Mouth ulceration	0	11 (2.6)
Stomatitis	0	10 (2.3)

*m* number of events, *n* number of patients, *TEAE* treatment-emergent adverse event

^a^≥ 5% of patients in either study phase

^b^Relationship to study drug classified as ‘possible’, ‘probable’ or ‘definite’

^c^≥ 1% of total events (dose-titration phase, N = 657; long-term safety phase, N = 1966) in either study phase

^d^Percentage of total number of events (dose-titration phase, N = 657; long-term safety phase, N = 1966)

^e^TEAEs with missing severity were considered severe

^f^> 2 patients in either study phase

^g^≥ 2% of patients in either study phase

##### TEAEs during dose-titration phase

During the dose-titration phase, 51.7% of patients experienced a total of 657 TEAE events. The most frequently reported TEAEs (≥ 5% of patients) were nausea (14.9%), yawning (9.6%), somnolence (6.7%) and headache (5.1%). TEAEs considered to be possibly, probably or definitely related to study drug were experienced by 38.5% of patients. The most frequently reported study drug-related TEAEs (≥ 1.5% of the total 657 TEAE events reported) were nausea (11.0%), yawning (10.5%), somnolence (7.2%), dizziness (5.0%), fatigue (3.5%) and dyskinesia (2.0%). The majority of the 657 TEAE events reported were of mild (80.1%) or moderate (17.4%) intensity, 2.6% being of severe intensity; the severe events were experienced by 3.1% of patients. Serious TEAEs were experienced by 1.3% of patients (n = 6). No serious TEAEs were reported by more than one patient. The six serious TEAEs were atrial fibrillation, drowning, sternal fracture, glioblastoma, bladder neck obstruction and deep vein thrombosis. One patient experienced a serious TEAE that was considered to be related to study drug (atrial fibrillation), which led to discontinuation of the study drug and subsequently resolved. One patient (0.2%) died due to a TEAE (drowning), but this was considered to be not related to study drug. TEAEs leading to study drug withdrawal were experienced by 6.7% of patients, including nausea (1.8%) and dizziness (1.3%). TEAEs of special interest were experienced by 26.9% of patients and the most frequently reported (≥ 5% of patients) were somnolence (6.7%) and dizziness (6.0%). TEAEs commonly associated with dopaminergic agents included orthostatic hypotension (2.9%), dyskinesia (2.7%), vomiting (1.6%) and hypotension (0.9%).

##### TEAEs during long-term safety phase

During the long-term safety phase, 85.7% of patients experienced a total of 1966 TEAE events. The most frequently reported TEAEs (≥ 10% of patients) were nausea (21.4%) and falls (10.3%). TEAEs considered to be possibly, probably or definitely related to study drug were experienced by 65.3% of patients. The most frequently reported related TEAEs (≥ 1.5% of the total 1966 TEAE events reported) were nausea (6.0%), stomatitis (1.8%), lip swelling (1.8%), dizziness (1.6%), oral mucosal erythema (1.6%) and mouth ulceration (1.6%). The majority of the 1966 TEAE events reported were of mild (58.1%) or moderate (36.2%) intensity, 5.6% being of severe intensity; the severe events were experienced by 13.4% of patients. Serious TEAEs were experienced by 13.6% of patients, the most frequently reported (≥ 1% of patients) being pneumonia (1.2%) and falls (1.2%). Four patients experienced serious TEAEs that were considered to be related to study drug (hypotension and syncope, n = 1; dysphagia and dyspnoea, n = 1; psychotic disorder, n = 1; dopamine dysregulation syndrome, n = 1). Study drug was discontinued in three of these patients and the TEAEs subsequently resolved; in the fourth patient, the event (dopamine dysregulation syndrome) resolved without requiring further action. Seven patients (1.6%) died due to TEAEs but none of the deaths was considered to be related to study drug (pneumonia, n = 2; cardio-respiratory arrest and pneumonia, n = 1; pneumonia aspiration, n = 1; myocardial infarction, n = 1; drowning, n = 1; sepsis, n = 1 [all considered ‘not related’ except sepsis, which was considered ‘unlikely related’]). TEAEs leading to study drug withdrawal were experienced by 34.0% of patients. The TEAEs that most frequently led to study drug withdrawal (≥ 2% of patients) were nausea (5.4%), lip swelling (4.5%), mouth ulceration (2.6%) and stomatitis (2.3%). TEAEs of special interest were experienced by 62.9% of patients and serious TEAEs of special interest were experienced by 7.3% of patients. Serious TEAEs of special interest experienced by more than two patients were fall (n = 5; 1.2%) and acute myocardial infarction (n = 3; 0.7%). Overall, 24.9% of patients experienced TEAEs of special interest leading to study drug withdrawal. The TEAEs of special interest that most frequently led to study drug withdrawal (≥ 2% of patients) were lip swelling (4.5%), mouth ulceration (2.6%) and stomatitis (2.3%). TEAEs commonly associated with dopaminergic agents included vomiting (4.7%), orthostatic hypotension (3.8%), hallucinations (2.6%) and hypotension (1.2%).

##### Oropharyngeal TEAEs

Oropharyngeal TEAEs were mapped into clinically relevant clusters (oropharyngeal oedema; oropharyngeal inflammation/erythema; oropharyngeal discolouration; oropharyngeal infections; oropharyngeal mass/neoplasm; oropharyngeal numbness/changes in sensation; oropharyngeal pain; oropharyngeal ulcerations; alterations in taste; salivary complaints and oral dryness; dental complaints; trauma; and other [oropharyngeal TEAEs that did not map to one of the other categories]).

During the dose-titration phase, 10.7% of patients experienced oropharyngeal TEAEs and one patient (0.2%) experienced an oropharyngeal TEAE that led to study drug withdrawal (dysgeusia). The most frequently affected clusters (≥ 2% of patients) were oropharyngeal inflammation/erythema (4.0%) and oropharyngeal ulcerations (2.4%). At the individual TEAE level, the most frequently reported oropharyngeal TEAEs (≥ 1% of patients) were oral mucosal erythema (3.8%) and dysgeusia (1.1%).

During the long-term safety phase, 39.7% of patients experienced oropharyngeal TEAEs and 18.8% of patients experienced oropharyngeal TEAEs that led to study drug withdrawal. The most frequently affected clusters (≥ 10% of patients) were oropharyngeal ulcerations (18.8%), oropharyngeal pain (11.5%) and oropharyngeal oedema (11.3%). The clusters most frequently associated with study drug withdrawal were oropharyngeal ulcerations (7.0%), oropharyngeal oedema (6.8%) and oropharyngeal pain (5.2%). At the individual TEAE level, the most frequently reported oropharyngeal TEAEs (≥ 5% of patients) were oral mucosal erythema (7.0%), lip swelling (6.3%), mouth ulceration (5.9%) and stomatitis (5.4%). The oropharyngeal TEAEs that most frequently led to study drug withdrawal (> 2% of patients) were lip swelling (4.5%), mouth ulceration (2.6%) and stomatitis (2.3%).

#### Physical examinations, including oropharyngeal cavity examinations

Generally, oropharyngeal cavity examination findings (including examination of the inside of the right and left cheeks, inside of the upper and lower lips, and surface and under the tongue) were minimal, with > 97% of patients presenting with no abnormalities in a given tissue location at a given visit.

#### Clinical laboratory parameters

Haematology, chemistry and urinalysis parameters remained stable throughout the study period, with the exception of vitamin B6 levels, which were elevated post-dose in 51.4%, 35.0%, 37.8% and 36.8% of patients at weeks 12, 24, 36 and 48, respectively, and in 20.7% of patients at last assessment. Such increases are expected since pyridoxine is an excipient included in the SL-APO formulation.

#### Vital signs, including orthostatic effects

Vital signs remained stable throughout the study and were similar during the dose-titration and long-term safety phases. There was no difference in the incidence of orthostatic hypotension during the dose-titration (35.4%) and long-term safety (34.5%) phases, and, during both phases, the proportion of patients with orthostatic hypotension was generally similar between the pre-dose and 60-min post-dose measurements. Few patients experienced TEAEs of orthostatic hypotension (2.9% during dose-titration phase; 3.8% during long-term safety phase).

#### Impulse control disorders (QUIP-RS)

The proportions of patients with a given impulse control disorder (gambling, sex, buying, eating, hobbyism, punding and medication use; as assessed using the QUIP-RS) remained stable during the course of the study. Mean total QUIP-RS and total impulsive control disorder scores also remained relatively stable over the course of the study (Supplementary Figure S1).

### Efficacy

**Fig. 4:**
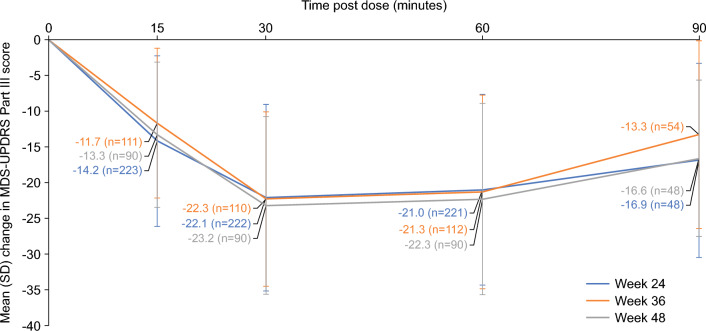
Reduction in MDS-UPDRS Part III score 15, 30 and 60 min post-SL-APO dosing after 24, 36 and 48 weeks (full analysis set). *MDS-UPDRS* Movement Disorder Society-sponsored Unified Parkinson’s Disease Rating Scale, *SD* standard deviation, *SL-APO* apomorphine sublingual film

Mean (SD) changes in MDS-UPDRS part III score 15, 30, 60 and 90 min post-SL-APO dosing at weeks 24, 36 and 48, respectively, are presented in Fig. 4. Clinically meaningful reduction in MDS-UPDRS part III score (based on published data [29]) was observed as soon as 15 min following administration of SL-APO, with peak effects observed approximately 30 min post-dose and sustained up to 90 min post-dose (the last time point measured). These results were consistent over 48 weeks (Fig. 4).

**Fig. 5:**
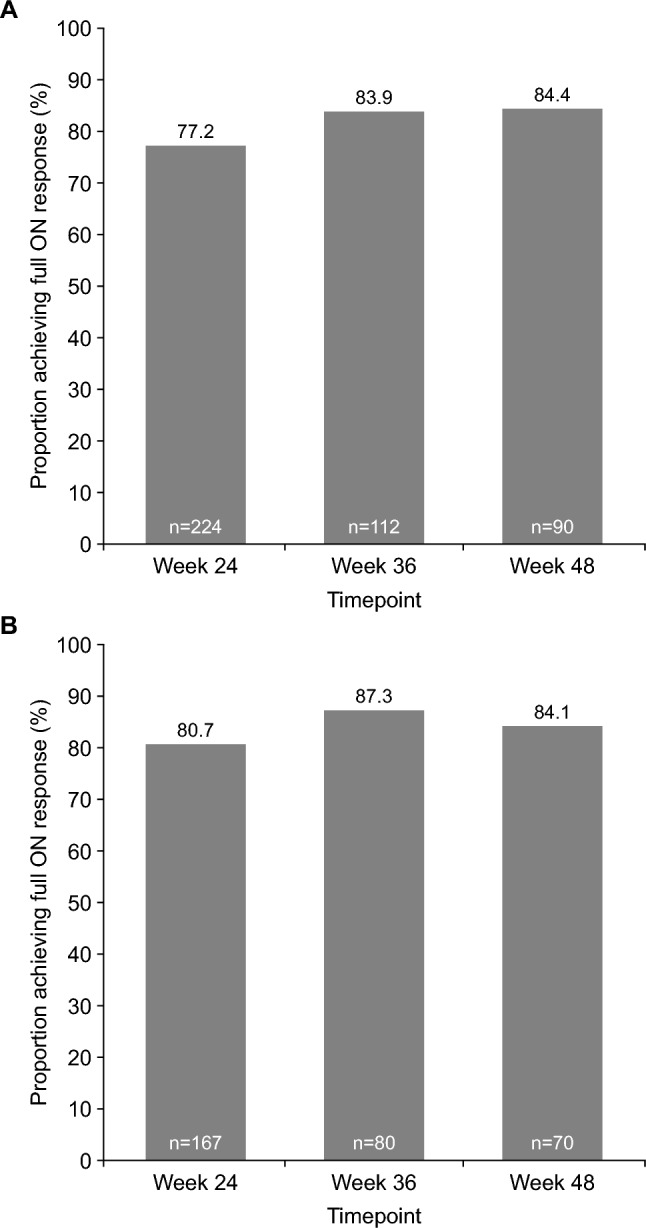
Achievement of self-rated full ON response within 30 min post-SL-APO dosing at weeks 24, 36 and 48 by **A** clinic visit assessment or **B** home dosing and response diary entry (full analysis set). *SL-APO* apomorphine sublingual film

At weeks 24, 36 and 48, self-rated full ON was achieved within 30 min post-dose in > 77% of SL-APO dosing instances, as assessed at clinic visits and as recorded in home-dosing and response diary entries in the 2 days prior to clinic visits (Fig. 5). For the clinic visit assessments, most patients continued to show a full ON response up to 60 min post-dose and approximately 50% of patients continued to show a full ON response up to 90 min post-dose (data not shown).

**Fig. 6:**
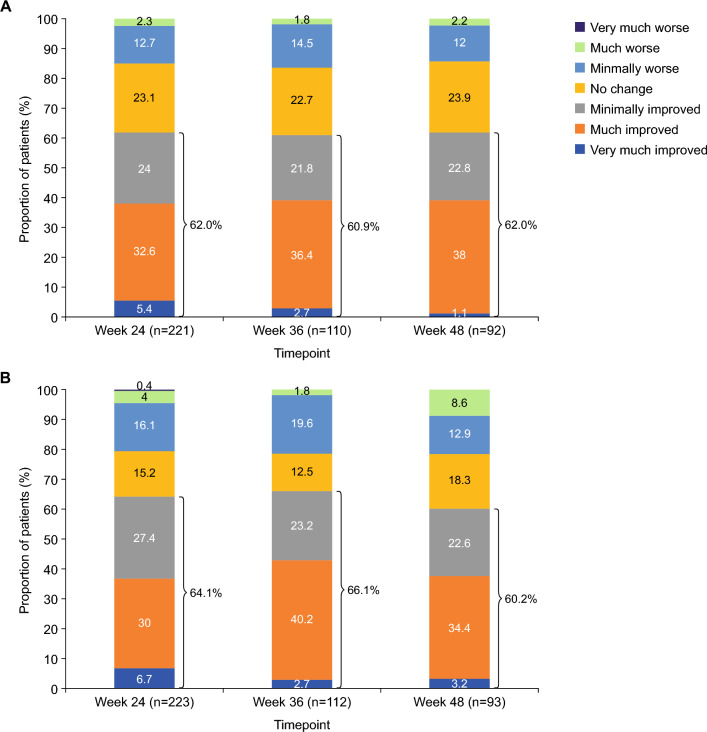
Proportions of patients rated as ‘very much improved’, ‘much improved’, ‘minimally improved’, ‘no change’, ‘minimally worse’, ‘much worse’ or ‘very much worse’ post-SL-APO dosing at weeks 24, 36 and 48 using **A** Clinical Global Impression of Improvement and **B** Patient Global Impression of Improvement (full analysis set). *SL-APO* apomorphine sublingual film

At weeks 24, 36 and 48, > 60% of patients were rated as having improved (i.e. ‘very much improved’, ‘much improved’ or ‘minimally improved’) post-SL-APO dosing, as assessed using both the CGI-I and PGI-I (Fig. 6).

**Fig. 7:**
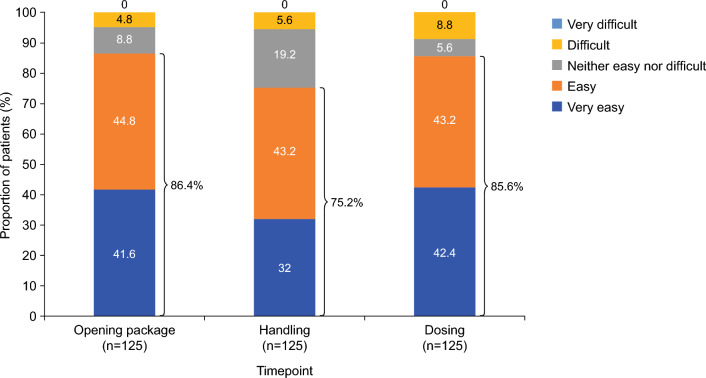
Ease of Use Questionnaire: proportions of patients who rated ‘opening the package’, ‘handling’ and ‘dosing’ as ‘very easy’, ‘easy’, ‘neither easy nor difficult’, ‘difficult’ or ‘very difficult’ (full analysis set)

The Ease of Use Questionnaire assessed patients’ ability to use SL-APO in terms of ‘opening the package’, ‘handling’ the product and ‘dosing’ the product on a five-point scale (‘very easy’, ‘easy’, ‘neither easy nor difficult’, ‘difficult’, ‘very difficult’). The proportion of patients who rated ease of use as ‘very easy’ or ‘easy’ was 86.4% for opening the package, 75.2% for handling, and 85.6% for dosing (Fig. 7). No patient rated any of these tasks as ‘very difficult’.

## Discussion

The objective of this phase 3, multicentre, open-label study was to assess the safety, tolerability and efficacy of SL-APO when used as an on-demand treatment for OFF episodes over the long term (mean duration of exposure, 294 days). SL-APO was shown to be generally well tolerated, and the safety profile observed in the current study was consistent with findings of previous SL-APO studies [24, 27, 28, 30], with no new or unexpected safety signals emerging with long-term use. During the dose-titration phase, the most frequent drug-related TEAEs (≥ 5% of events) were nausea, yawning, somnolence and dizziness, but during the long-term safety phase, the only drug-related TEAE representing > 2% of events was nausea (6.0%). Although nausea is common in PD patients initiating dopamine agonists, a *post-hoc* analysis of this study’s dose-titration phase has shown that prophylactic treatment with an antiemetic was not necessary for a subset of patients titrating to their effective and tolerable dose, as separately published [31]. It is recommended that if a prophylactic antiemetic, such as trimethobenzamide and domperidone, is considered medically warranted during SL-APO titration in clinical practice, the lowest effective dose should be utilised and discontinued as soon as possible [25]. Serious related TEAEs were experienced by < 1% of patients and all the events subsequently resolved. Most TEAEs (> 90% of all events) were of mild or moderate intensity. During the long-term safety phase, 34% of patients experienced TEAEs leading to discontinuation, and the TEAEs most frequently leading to discontinuation were nausea, lip swelling, mouth ulceration and stomatitis. Approximately 40% of patients experienced oropharyngeal TEAEs during the long-term safety phase and these were the TEAEs that most frequently resulted in treatment discontinuation (19% of patients); however, it is noteworthy that 21% of patients experienced oropharyngeal TEAEs but did not discontinue treatment because of these adverse reactions. Oropharyngeal TEAEs were not limited to the sublingual area (where the film was placed), but most either resolved spontaneously or after treatment discontinuation. It is important to note that, following discontinuation due to oropharyngeal TEAEs, it is recommended that SL-APO treatment is not subsequently reintroduced, since oral adverse reactions may recur and be more severe than the initial reaction [25]. Clinicians should be vigilant for the development of oropharyngeal adverse reactions (such as oral mucosal erythema, mouth ulceration, lip swelling and stomatitis) in real-world practice to assess possible discontinuation. The accretion of real-world experience with SL-APO may fuel a consensus on how to mitigate and manage oropharyngeal adverse reactions and identify patient factors associated with predisposition for such adverse reactions, which could potentially result in the development of prophylactic treatment. TEAEs were the most common reason for discontinuation during both phases of the study. The rate of discontinuation was higher in de novo than in rollover patients, particularly during the dose-titration phase, which was likely due to rollover patients’ previous enrolment in other studies with SL-APO. However, the rate of discontinuation during dose titration among de novo patients was lower versus the rate during dose titration in the phase 3 study CTH-300 (17.3% vs. 22.7% for overall discontinuation; 6.8% vs. 8.5% for discontinuation due to AEs; 3.3% vs. 7.8% for discontinuation due to lack of efficacy) [24]. There were no notable laboratory or vital sign findings in either phase of the study, and there was no increase in impulse control behaviours with long-term exposure to SL-APO. Orthostatic hypotension (which included the medical dictionary for regulatory activities [MedDRA] terms ‘hypotension orthostatic asymptomatic’, ‘hypotension postural aggravated’, ‘postural hypotension’, ‘hypotension orthostatic’, ‘hypotension postural’ and ‘hypotension orthostatic symptomatic’) affected approximately one-third of patients during both phases of the study, but its incidence was generally similar pre-dose and 60-min post-dose, and < 4% of patients experienced TEAEs of orthostatic hypotension.

The efficacy of SL-APO in managing motor fluctuations was supported by multiple indicators, including clinically meaningful reductions in MDS-UPDRS part III score, high levels of patient- and investigator-rated full ON response within 30 min of dosing (as well as at 60 and 90 min), and improvements in the CGI-I and PGI-I over time. A clinically meaningful reduction from pre-dose value in MDS-UPDRS part III score [29] was observed as soon as 15 min following administration of SL-APO. This was sustained up until the last time point measured (90 min post-dose), and these results were consistent over 48 weeks. Patient-rated full ON was achieved within 30 min in over three-quarters of SL-APO dosing instances, and these results were again consistent over 48 weeks. At all time points up to week 48, > 60% of patients were rated as having improved on both the CGI-I and PGI-I. Assessment of ease of use indicated that the majority of patients experienced little or no difficulty with self-administering SL-APO at home. Taken together, the findings from this open-label study are consistent with those from double-blind trials and demonstrate that SL-APO is generally well tolerated and efficacious in improving motor fluctuations in patients with OFF episodes over the long term (approximately 1 year).

The current non-randomised study can be considered in the context of other recent studies. First, an open-label, randomised, crossover study assessed SL-APO versus subcutaneous apomorphine in patients with PD and OFF episodes (study CTH-302) [28]. Following dose optimisation and a washout period, patients received 4 weeks of treatment with their optimised dose of SL-APO or subcutaneous apomorphine, followed by a further washout period and 4 weeks of crossover treatment [28]. There was no significant difference between the treatments for change from pre-dose to 90 min post-dose in MDS-UPDRS part III score at week 4 (primary endpoint) [28]. However, results from the Treatment Preference Questionnaire demonstrated that 72.2% of patients preferred SL-APO to subcutaneous apomorphine/no preference (p = 0.0002), and results from the Treatment Satisfaction Questionnaire for Medication showed greater satisfaction with SL-APO versus subcutaneous apomorphine for convenience (mean score, 73.7 vs. 53.5) and global satisfaction (mean score, 63.9 vs. 57.6) [28]. Both treatments were well tolerated and their safety profiles were generally comparable; for example, the incidence of nausea during open-label treatment was 14.1% for SL-APO versus 15.7% for subcutaneous apomorphine [28]. Second, a discrete choice experiment that evaluated preferences for select attributes of theoretical on-demand treatments for OFF episodes among 300 US adults with PD found that the respondents preferred a theoretical dissolvable sublingual film over a theoretical inhaled medicine or theoretical injected medicine, regardless of whether these three modes of administration were associated with AEs or not [32]. For modes with AEs, respondents preferred a dissolvable sublingual film that may cause mouth or lip sores to an inhaled medicine that may cause cough or mild respiratory infection, and they also preferred an inhaled medicine with a risk of cough or mild respiratory infection over an injected medicine with possible injection-site reactions [32].

The availability of on-demand treatments for OFF episodes has led to a call for a ‘treatment paradigm shift’ in terms of considering these treatments earlier and throughout the disease course of PD [8]. The rationale for this recommendation is based on the persistence of OFF episodes despite adjunctive treatment, greater understanding of dysphagia/gastrointestinal dysmotility and variability of oral levodopa absorption, and the impact of OFF episodes on patients’ daily activities and quality of life [8]. The authors of this recommendation concluded that on-demand treatments can help to empower patients to recognise and rapidly treat OFF episodes and should therefore be routinely incorporated into shared clinical decision-making [8]. More recently, a modified Delphi panel was employed by a group of PD experts to develop consensus on the use of on-demand treatments for OFF episodes [19]. The experts agreed that on-demand treatment is appropriate for many patients with OFF episodes, including those whose OFF episodes have a significant functional impact on their lives, or follow a pattern of delayed ON, dose failure, or morning OFF; those who use higher doses of levodopa in addition to on-extender treatments; and those who experience treatment-related side effects with levodopa and/or on-extender treatments [19]. Overall, the experts highlighted that on-demand treatment could be particularly beneficial for individuals whose OFF episodes significantly affect their quality of life [19].

This study has acknowledged limitations. Its open-label, non-randomised design could potentially have led to an overestimation of treatment efficacy. Additionally, the absence of formal statistical testing limits the strength of conclusions drawn from the descriptive summaries. However, the observed change in MDS-UPDRS part III far exceeded the minimal clinically important difference [29]. The study population was diverse, including patients from both the US and Europe, with variations in concomitant medication and disease duration. While this diversity enhances the applicability of the results to real-world settings, it introduces potential confounding factors. Despite geographical and clinical variation in the study population, 96% of patients were White and the lack of racial diversity therefore limits the external validity of the findings to non-White patient groups [33]. The inclusion of rollover patients might have influenced safety outcomes and patient withdrawal rates. The overall rate of discontinuation was higher among de novo patients than rollover patients during the dose-titration phase (17% vs. 5%), as was the rate of discontinuation due to AEs (7% vs. 2%). However, the overall rate of discontinuation was similar among de novo patients and rollover patients during the long-term safety phase (61% vs. 64%) and so was the rate of discontinuation due to AEs (29% vs. 24%). It is also noteworthy that the rate of discontinuation due to withdrawal of consent by patients was high (18% during the long-term safety phase), and it is possible that some of those who withdrew consent did so due to a perceived lack of efficacy. However, the most common reason for discontinuation during both phases of the study was AEs, including the observation that during the long-term safety phase (when most discontinuations occurred), 28.0% of patients discontinued due to AEs. Although the mean number of total daily OFF episodes was 3.9, patients administered treatment approximately 1.7 times daily, suggesting that patients decided whether to administer SL-APO immediately or wait for their fixed-regimen dose based on the severity of each OFF episode. It is possible that patients considered the risk/benefit trade-off between tolerability and efficacy when deciding whether to use SL-APO for a particular OFF episode. It is also possible that the use of SL-APO was affected by difficulties in identifying OFF episodes, highlighting the importance of patient education in terms of both identifying OFF episodes and becoming confident in using an on-demand treatment for OFF episodes. This study is also limited because it did not assess the impact of SL-APO intake on non-motor fluctuations. Virtually all individuals with PD experience non-motor symptoms and many experience non-motor fluctuations [34, 35]. Future research is therefore required to investigate the potential utility of SL-APO as an on-demand treatment for severe non-motor fluctuations. Further research is also required to clarify whether SL-APO is effective in treating both predictable and unpredictable OFF episodes, since this was not assessed in the current study. Finally, newly enrolled patients up-titrated exclusively in the clinic, differing from the home titration approach used in the CTH-302 study. This variation from real-world practice might impact safety during titration, although a comparison of SL-APO dose optimisation methods between CTH-300 (in-clinic titration) and CTH-302 (home titration) demonstrated comparable safety levels [36]. Additional *post-hoc* analyses are currently ongoing to assess the potential impact of concomitant use of dopamine agonists on outcomes with SL-APO.

In summary, this study adds to the available evidence for SL-APO by demonstrating that it is generally well tolerated and efficacious over the long term as an on-demand treatment for OFF episodes in PD patients. The ease of use of SL-APO will be beneficial to patients afraid of more invasive therapies and the sublingual formulation addresses challenges associated with other on-demand treatment options [37]. SL-APO therefore represents a valuable effective on-demand treatment for OFF episodes in patients with PD over the long term.

### Supplementary Information

Below is the link to the electronic supplementary material.

Supplementary file1 (DOCX 93 KB)

## Data Availability

Access to de-identified participant data will be provided after a research proposal is submitted online (https://vivli.org) and receives approval from the Independent Review Panel and after a data-sharing agreement is in place. Access will be provided for an initial period of 12 months after approval of the data-sharing request, but an extension can be granted, when justified, for up to an additional 12 months.
