# Supplementary material for: Long-term safety, tolerability and efficacy of apomorphine sublingual film in patients with Parkinson’s disease complicated by OFF episodes: a phase 3, open-label study
Source: J Neurol. 2024 Mar 28;271(6):3554–70. doi: 10.1007/s00415-024-12323-2 (PMC11136727; doi:10.1007/s00415-024-12323-2)
Supplement: Supplementary file 1 — Supplementary file1 (DOCX 93 KB) [file 415_2024_12323_MOESM1_ESM.docx]

**Long-term safety, tolerability and efficacy of apomorphine sublingual film in patients with Parkinson’s disease complicated by OFF episodes: A Phase 3, open-label study**

**Jan Kassubek^1,2^, Stewart A. Factor^3^, Ernest Balaguer^4^, Johannes Schwarz^5^, K. Ray Chaudhuri^6^, Stuart H. Isaacson^7^, Stacy Wu^8^, Carmen Denecke Muhr^9^, Jaime Kulisevsky^10,11^**

^1^*Department of Neurology, University Hospital Ulm, Ulm, Germany; ^2^German Centre for Neurodegenerative Diseases, Ulm, Germany (ORCID 0000-0002-7106-9270); ^3^Jean and Paul Amos Parkinson’s Disease and Movement Disorder Program, Emory University School of Medicine, Atlanta, Georgia, USA (ORCID 0000-0002-0449-973X); ^4^Hospital Universitari General de Catalunya, Barcelona, Spain (ORCID 0000-0001-9258-1735); ^5^Department of Geriatrics, Kreisklinik Ebersberg, Ebersberg, Germany (ORCID 0009-0002-8314-5573); ^6^King's College London, Department of Neurosciences, Institute of Psychiatry, Psychology & Neuroscience and Parkinson's Foundation Centre of Excellence, King's College Hospital, London, UK (ORCID 0000-0003-2815-0505); ^7^Parkinson’s Disease and Movement Disorders Center of Boca Raton, Boca Raton, Florida, USA (ORCID 0000-0002-9914-5706); ^8^Sumitomo Pharma America, Inc., Marlborough, Massachusetts, USA; ^9^BIAL – Portela & C^a^ S.A., Porto, Portugal; ^10^Hospital de la Santa Creu i Sant Pau, Barcelona, Spain; ^11^Universitat Autònoma de Barcelona and CIBERNED, Madrid, Spain (ORCID 0000-0003-4870-1431)

***Corresponding author:**

Jan Kassubek,

Department of Neurology,

University Hospital Ulm,

Oberer Eselsberg 45,

D 89081 Ulm,

Germany

Email: [jan.kassubek@uni-ulm.de](mailto:jan.kassubek@uni-ulm.de)

**Journal of Neurology**

**Supplementary Information**

**METHODS**

**Study design**

*Details of central Institutional Review Boards/Independent Ethics Committees:* USA, WCG IRB (registration # IRB00000533); Austria, Ethikkommission der Medizinischen Universität Innsbruck; Germany, Ethikkommission der Universität Ulm; Italy, AIFA – Agenzia Italiana del Farmaco, Ethics Committee Coordination Centre; Spain, Agencia Española de Medicamentos y Productos Sanitarios (AEMPS); United Kingdom, NHS – Health Research Authority.

**Patient population**

*Additional eligibility criteria:* For both the *de novo* and rollover groups, female patients of childbearing potential had to agree to be sexually abstinent or to use a highly effective method of birth control from first study medication administration until 90 days after final medication administration, and male patients either had to be sexually sterile, agree to be sexually abstinent, or agree to use a double-barrier method of birth control from first study medication administration until 90 days after final medication administration. All patients also had to be able and willing to comply with all study procedures and provide written informed consent. *De novo* participants were excluded from participation if they had a history of drug/alcohol dependency within the previous 12 months, a history of malignancy within 5 years prior to the screening visit (except for adequately treated basal cell carcinoma or squamous cell skin cancer, or in situ cervical cancer), dementia that precluded providing informed consent or interfered with study participation, a clinically significant medical, surgical or laboratory abnormality (Investigator’s opinion), or suicidal ideation withing 1 year prior to screening visit 2 (as assessed using the Columbia Suicide Severity Rating Scale [C-SSRS] or evidenced by attempted suicide in the previous 5 years). Rollover patients were excluded if they had any major psychiatric disorder or any disorder requiring ongoing treatment that would have made study participation unsafe or compliance difficult (Investigator’s opinion), any clinically significant medical, surgical or laboratory abnormality that would have made study participation unsafe or compliance difficult (Investigator’s opinion), or current suicidal ideation (as assessed using the C-SSRS). For both the *de novo* and rollover groups, female patients were excluded if they were pregnant or lactating.

**Supplementary Figure S1. QUIP-RS: Total ICD Score and Total QUIPS-RS Score at baseline and weeks 24, 36 and 48 (Full Analysis Set).** ICD, Impulse Control Disorder; QUIP-RS, Questionnaire for Impulsive-Compulsive Disorders in Parkinson’s Disease-Rating Scale; SD, standard deviation

**
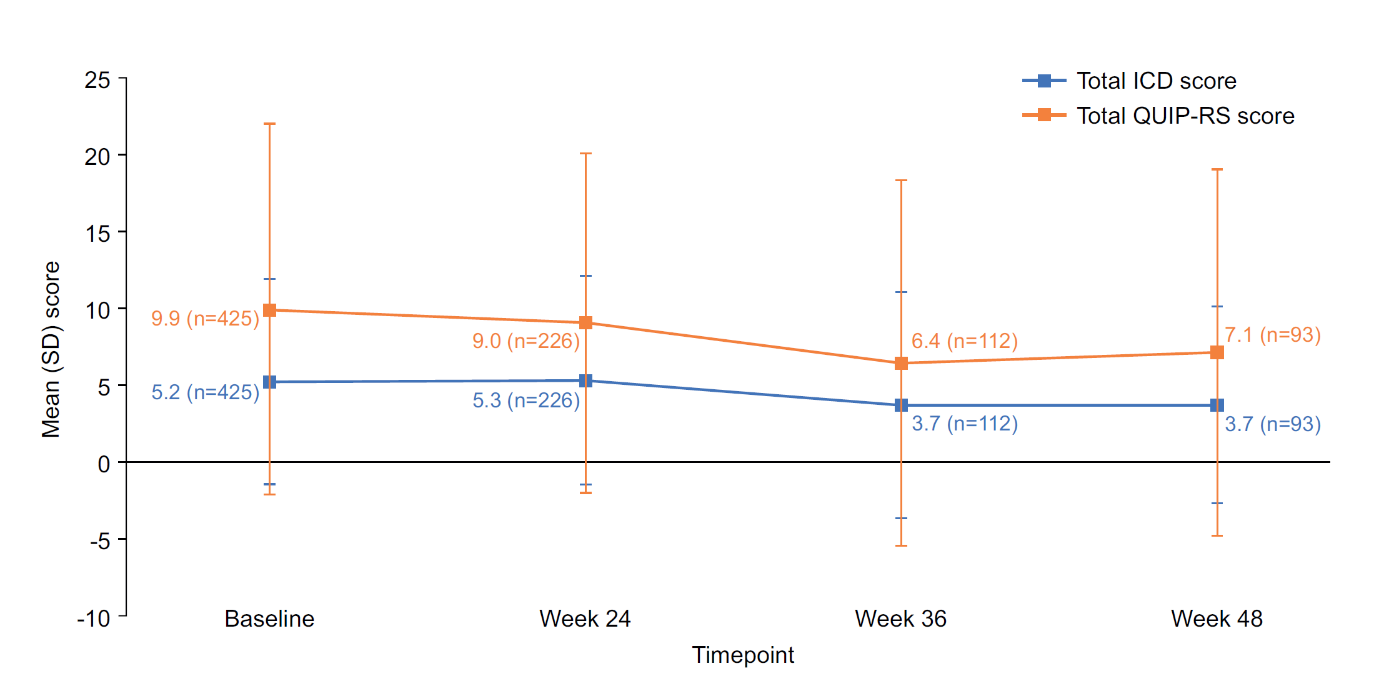
**
